# A review on diabetes and oral cancer: Molecular links and implications

**DOI:** 10.22099/mbrc.2024.51225.2042

**Published:** 2025

**Authors:** Jayaseelan Vijayashree Priyadharsini, Anitha Pandi

**Affiliations:** Clinical Genetics Lab, Centre Cellular and Molecular Research (The Blue Lab), Saveetha Dental College & Hospital, Saveetha Institute of Medical and Technical Sciences [SIMATS], Saveetha University, Chennai, Tamil Nadu, India

**Keywords:** Diabetes, Oral Cancer, Inflammation, Hyperglycemia

## Abstract

Diabetes mellitus has been linked to an increased risk of oral cancer, with hyperglycemia and chronic inflammation contributing to malignant transformation. Accumulating evidence has highlighted the role of specific genes and biomarkers associated with the process. While hyperglycemia accelerates cancer progression, Metformin, an anti-diabetic medication, is found to reduce the recurrence. Future research should focus on understanding molecular mechanisms, developing early diagnostic tools, and assessing the impact of glycemic control in managing potentially oral malignant lesions in diabetic patients.

## INTRODUCTION

Diabetes mellitus (DM) is a global concern with a progressively increasing incidence rate. The International Diabetes Federation has projected that the prevalence of diabetes, which stood at 10.5% in 2021, is expected to rise to 11.3% by 2030 and 12.2% by 2040. This increase is expected to be particularly pronounced in low and middle-income countries [1]. DM, a metabolic disorder and a co-morbid condition associated with various other diseases, has recently been a potential risk factor for a few solid malignancies of the pancreas, colon, breast, liver, and endometrium [2, 3]. 

It is also reported that the mortality rate of cancer patients with diabetes is relatively higher in comparison to non-diabetic patients. In addition, the complications related to renal, cardiovascular, cerebrovascular diseases, and chronic infections in diabetic patients markedly influenced their cancer therapy. These complications substantially lowered the benefits of the treatment and eventually led to a shorter survival rate [4]. 

A systematic review and meta-analysis conducted by Ramos-Garcia and colleagues demonstrated an increased prevalence of potentially malignant lesions of the oral cavity and oral cancer among patients with DM [5]. Despite the evidence accumulated, the genes involved in DM-mediated inflammatory pathways or other networks leading to the malignant transformation of cells have not been discussed comprehensively. Hence, this review aims to reveal the interconnected pathways and processes common to diabetes and oral cancer in terms of immunological mechanisms, metabolic pathways, and risk variants common to both disease conditions.

### Immunogenetics: crosstalk between diabetes and cancer:

 Inflammation is regarded as one of the critical hallmarks of cancer. The phenotypes of diabetes and cancer have a common pathway mediated through inflammatory markers. Numerous factors, such as infections, autoimmune conditions, environmental exposure, habits like smoking and alcoholism, can trigger inflammation. Periodontitis (PD), a chronic inflammatory condition affecting the soft tissues surrounding the teeth [6], has been found to modulate the tumor microenvironment. Pathogenic microorganisms such as *Fusobacterium *and *Porphyromonas gingivalis,* have been reported to play a critical in establishing chronic inflammation and tissue destruction [7]. Thus it has been proved that the presence of PD increases the risk of developing diabetes. A direct link between cancer and inflammation has also been reported, which is characterized by neutrophil invasion, matrix degradation, and the formation of invadopodia [8].

A recent study on *TREM1* (triggering receptor expressed on myeloid cells 1), a receptor of the immunoglobulin superfamily expressed on myeloid cells, indicated a relationship between chronic periodontal illness and type 2 diabetes mellitus [9]. Another study by Fan and team identified eight crucial genes that were immunologically related cross-talk genes, which were *C1QC* (Complement C1q subcomponent, C chain), *ABCD1* (ATP-Binding Cassette Sub-Family D Member 1), *NOS2* (Nitric Oxide Synthase 2), *PD1A4 *(PD-1 (Programmed Cell Death Protein 1) Auxiliary Protein 4), *IL1RN *(Interleukin 1 Receptor Antagonist), *ALOX15* (Arachidonate Lipoxygenase 15), *CSE1L* (Chromosome Segregation 1-Like) and *PSMC4* (Proteasome 26S Subunit ATPase 4) related to OSCC (Oral Squamous Cell Carcinoma) and T2D (type 2 diabetes mellitus). The receiver operating characteristic (ROC) curve analysis revealed that *ABCD1, C1QC, CSE1, *and *PSMC4* had a strong prediction effect among the eight genes [10].

### Glucose metabolism disorder and oral cancer:

 Studies have reported alterations in glucose metabolism at a very early stage during the development of oral malignancies. The significant upregulation of *GLUT1* or the solute carrier family 2 member 1 protein correlated well with the nodal status. In addition, the upregulation of GLUT1 presented with adverse outcomes influencing the survival of oral cancer patients [11]. A retrospective analysis conducted among Austrian patients with oral cancer revealed a significant difference in glucose metabolism among the cancer patients (59.9%) when compared to the control group (36.5%), thus proving a strong association between glucose metabolism disorder and oral cancer [12]. A similar kind of association study was conducted in Korean populations with around 9,598,085 individuals over 20 years of age. They observed that subjects with metabolic syndrome exhibited an increased susceptibility towards the development of oral and laryngeal cancer. Interestingly, this association was found to remain significant even after adjusting for smoking and consumption of alcohol [13]. 

Vegh and the team pursued an analysis to demonstrate the relationship between diabetes mellitus and cancer in the Hungarian population. They observed 54.4% of oral cancer patients with elevated levels of glucose in the blood, of which 61.1% and 4.7% had T2D and type 1 diabetes (T1D), respectively. About 34.2% of the patients had impaired glycemic index [14]. Hu and colleagues provided strong evidence on the progression of OSCC in T2D patients. The study documented a lower 5-year survival rate in patients with OSCC with T2D compared to those without T2D. Elevated glucose levels led to increased proliferation of cancer cells and reduced apoptosis and migration. The upregulation of biomarkers such as Ki67 (antigen Kiel 67) and downregulation of BRIP1 (BRCA1-interacting protein 1), E-cadherin, and cleaved caspase-3 in diabetic patients were suggestive of acceleration of oral cancer progression in conditions of hyperglycemia [15].

### Genetic variants associated with DM and oral cancer:

Human genetic variations such as single nucleotide polymorphisms or mutations have a profound effect on the development of diseases. These variants either individually or collectively affect the phenotypes, causing susceptibility or resistance towards a specific disease condition. Common genetic variants of candidate genes such as *TCF7L2* (transcription factor 7 like 2), *PPARG* (encoding peroxisome proliferator-activated receptor γ), *HNF1B* (encoding hepatocyte nuclear factor 1 homeobox B), *JAZF1* (encoding JAZF zinc finger 1), *KLF14* (encoding Kruppel-like factor 14) were reported to be associated with different cancer types including cancer of oral cavity, breast, colon, pancreatic, liver, prostate and many more [16].

A recent study on the outcome of LDL (low-density lipoprotein) receptor-related protein 1B polymorphism in diabetic patients with oral cancer revealed a strong association of the minor allele of rs10496915 with tumor size in DM patients. Furthermore, the heterozygous genotype and the combination of minor alleles of rs6742944 were associated with lymph node metastasis and advanced clinical stages. These observations also provided clues on how the genetic variations influence the cancer phenotypes in DM patients [17]. Salehi and the team presented contradictory yet surprising results on the link between oral cancer and DM. A machine learning approach was adopted to study the recurrence and survival of tongue squamous cell carcinoma patients. A lower recurrence rate and an improved two-year survival were observed in cancer patients with diabetes when compared to non-diabetic individuals [18]. One possible reason for this observation could be attributed to Metformin, an anti-diabetic drug usually prescribed for T2D, which has been reported to exhibit an anti-tumor effect [19]. 

Secondly, it can be hypothesized that some immune mechanisms activated during T2D could paradoxically suppress tumor growth or recurrence by triggering anti-tumor mechanisms [20]. Epigenetic mechanisms and genetic and environmental factors could act in concert to modulate the tumor's immune environment [21]. This understanding of the mechanisms underscores the importance of glycemic control in diabetes management for overall health and potentially reducing the risk of oral cancer development in susceptible individuals, where early interventions remain vital to mitigating the risks [22]. Additionally, knowledge about the genetic network can help to curate a hub of genes with prognostic and diagnostic significance linked to dysplastic changes in diabetic patients [23]. 

### Oral potentially malignant disorders and diabetes:

Oral potentially malignant disorders (OPMDs) have a high chance of malignant transformation to oral carcinoma precipitated by chronic inflammation and oxidative stress. Recently, the focus has shifted towards genomic and epigenomic landscapes common to both disease conditions. The amplification of *the EGFR* gene (epidermal growth factor receptor), its upregulation, and increased protein expression can be used to predict tumor progression significantly. A report by Wisniewski et al. demonstrated the critical role of fatty acid synthase (FASN) on the activation of EGFR in nicotine-treated oral dysplastic keratinocytes exposed to high glucose. This increased cell viability and migration in a FASN/EGFR-mediated pathway [24]. 

A similar study by Tao and colleagues revealed the role of amphiregulin (AREG) as a glucose-responsive ligand in oral dysplastic keratinocytes under hyperglycemic conditions. They identified that high glucose significantly increased glycolytic activity and activated EGFR, enhancing cell viability and migration. AREG emerged as the predominant ligand induced by high glucose, inhibiting migration, while treatment with AREG under normal glucose conditions elevated cell viability and glycolytic markers. These findings suggest AREG's potential role in hyperglycemia-associated oral tumor progression [25]. These studies not only aid in creating the genetic hub of candidate genes crucial in potentiating premalignancy conditions but also help us understand the key pathways and networks disrupted during disease progression.

## Conclusion

Collectively, future research should be focused on the methods to decipher the effect of DM control in the context of the management OPMDs and oral cancer by a) conducting longitudinal studies that would determine whether diabetes-related hyperglycemia causes the evolution of OPMDs to oral cancer, b) understanding different mechanisms such as insulin resistance, oxidative process, chronic inflammation related to tumor development among diabetic patients, c) establish and test biomarkers which intervene to transform OPMD in diabetic individuals, d) propose intervention studies to measure how these control strategies or the side effects of active control in patients with OPMDs lowers the risk of oral cancer, and finally, e) endocrinologists, oncologists and dentists should be involved in performing investigations focused on designing standard strategies for treating oral cancer in patients with diabetes.

## References

[1] Ye J, Wu Y, Yang S, Zhu D, Chen F, Chen J, Ji X, Hou K (2023). The global, regional and national burden of type 2 diabetes mellitus in the past, present and future: a systematic analysis of the Global Burden of Disease Study 2019. Front Endocrinol (Lausanne).

[2] Shahid RK, Ahmed S, Le D, Yadav S (2021). Diabetes and cancer: risk, challenges, management and outcomes. Cancers (Basel).

[3] Giovannucci E, Harlan DM, Archer MC, Bergenstal RM, Gapstur SM, Habel LA, Pollak M, Regensteiner JG, Yee D (2010). Diabetes and cancer: a consensus report. Diabetes Care.

[4] Xu W, Chen Z, Zhang L (2024). Impact of diabetes on the prognosis of patients with oral and oropharyngeal cancer: A meta-analysis. J Diabetes Investig.

[5] Ramos-Garcia P, Roca-Rodriguez MDM, Aguilar-Diosdado M, Gonzalez-Moles MA (2021). Diabetes mellitus and oral cancer/oral potentially malignant disorders: A systematic review and meta-analysis. Oral Dis.

[6] Gupta A, Singh N, Kumar A, Verma UP, Verma AK, Shyam H, Lal N, Kant S, Kumari A (2020). Differential expression of inflammatory responsive genes between chronic periodontitis and periodontally affected bronchiectasis patients. Mol Biol Res Commun.

[7] Ezhilarasan D, Varghese SS (2022). Porphyromonas gingivalis and dental stem cells crosstalk amplify inflammation and bone loss in the periodontitis niche. J Cell Physiol.

[8] Goertzen C, Mahdi H, Laliberte C, Meirson T, Eymael D, Gill-Henn H, Magalhaes M (2018). Oral inflammation promotes oral squamous cell carcinoma invasion. Oncotarget.

[9] Syed VA, Ramamurthy J (2024). Evaluation of TREM1 gene expression in stage II grade A periodontitis patients with type 2 diabetes mellitus: An ex-vivo study. J Clin of Diagn Res.

[10] Fan Y, Zhang J, Shi J, Chen L, Long J, Zhang S, Liu S (2022). Genetic cross-talk between oral squamous cell carcinoma and type 2 diabetes: The potential role of immunity. Dis Markers.

[11] Nakazato K, Mogushi K, Kayamori K, Tsuchiya M, Takahashi KI, Sumino J, Michi Y, Yoda T, Uzawa N (2019). Glucose metabolism changes during the development and progression of oral tongue squamous cell carcinomas. Oncol Lett.

[12] Remschmidt B, Pau M, Gaessler J, Zemann W, Jakse N, Payer M, Vegh D (2022). Diabetes mellitus and oral cancer: A retrospective study from Austria. Anticancer Res.

[13] Kim GJ, Han KD, Joo YH (2023). Association of metabolic syndrome with the risk of head and neck cancer: A 10-year follow-up study of 10 million initially healthy individuals. Cancers (Basel).

[14] Vegh A, Banyai D, Ujpal M, Somogyi KS, Biczo Z, Kammerhofer G, Nemeth Z, Hermann P, Payer M, Vegh D (2022). Prevalence of diabetes and impaired fasting glycemia in patients with oral cancer: A retrospective study in Hungary. Anticancer Res.

[15] Hu X, Wu J, Xiong H, Zeng L, Wang Z, Wang C, Huang D, Zhang T, Peng Y, Chen W, Xie K, Su T (2022). Type 2 diabetes mellitus promotes the proliferation, metastasis, and suppresses the apoptosis in oral squamous cell carcinoma. J Oral Pathol Med.

[16] Vincent EE, Yaghootkar H (2020). Using genetics to decipher the link between type 2 diabetes and cancer: shared aetiology or downstream consequence?. Diabetologia.

[17] Chen LC, Lo YS, Ho HY, Lin CC, Chuang YC, Chang WC, Hsieh MJ (2024). LDL Receptor-related protein 1B polymorphisms associated with increased risk of lymph node metastasis in oral cancer group with diabetes mellitus. Int J Mol Sci.

[18] Salehi AM, Wang L, Gu X, Coates PJ, Spaak LN, Sgaramella N, Nylander K (2024). Patients with oral tongue squamous cell carcinoma and co‑existing diabetes exhibit lower recurrence rates and improved survival: Implications for treatment. Oncol Lett.

[19] Hu X, Xiong H, Chen W, Huang L, Mao T, Yang L, Wang C, Huang D, Wang Z, Yu J, Shu Y, Xia K, Su T (2020). Metformin reduces the increased risk of oral squamous cell carcinoma recurrence in patients with type 2 diabetes mellitus: A cohort study with propensity score analyses. Surg Oncol.

[20] Peña-Romero AC, Orenes-Piñero E (2022). Dual effect of immune cells within tumour micro-environment: Pro- and anti-tumour effects and their triggers. Cancers (Basel).

[21] Muthumanickam P, Ramasubramanian A, Pandi C, Ramani P, Jayaseelan VP, Arumugam P (2024). The novel m6A writer methyltransferase 5 is a promising prognostic biomarker and associated with immune cell infiltration in oral squamous cell carcinoma. J Oral Pathol Med.

[22] Peppa M, Manta A, Mavroeidi I, Nastos C, Pikoulis E, Syrigos K, Bamias A (2023). Dietary approach of patients with hormone-related cancer based on the glycemic index and glycemic load estimates. Nutrients.

[23] Bs A, P A, As SG, A P, J VP (2024). Analysis of differentially expressed genes in dysplastic oral keratinocyte cell line and their role in the development of HNSCC. J Stomatol Oral Maxillofac Surg.

[24] Wisniewski DJ, Ma T, Schneider A (2021). Fatty acid synthase mediates high glucose-induced EGFR activation in oral dysplastic keratinocytes. J Oral Pathol Med.

[25] Ma T, Montaner S, Schneider A (2023). Glucose upregulates amphiregulin in oral dysplastic keratinocytes: A potential role in diabetes-associated oral carcinogenesis. J Oral Pathol Med.

